# Multiple myeloma revealed by spinal cord compression and herpes zoster in a 36-year-old Cameroonian

**DOI:** 10.11604/pamj.2013.16.21.2908

**Published:** 2013-09-18

**Authors:** Victor Sini, Tatuene Joseph Kamtchum, Callixte Kuate Tegueu, Vincent De Paul Djientcheu

**Affiliations:** 1 Department of neurology, Yaoundé Central Hospital, Cameroon; 2 Department of neurosurgery, Central Hospital of Yaoundé, Cameroon; 3 Department of neurology, Douala Laquintinie Hospital, Cameroon

**Keywords:** Multiple myeloma, plasmocytosis, spinal cord compression, young age, herpes zoster, Cameroonian

## Abstract

Multiple myeloma is a malignant plasma cell disorder occurring mostly in people above 60 years old. The authors describe a case of multiple myeloma in a 36-year-old patient revealed by spinal cord compression and Herpes zoster with a rapidly unfavourable outcome.

## Introduction

Multiple myeloma or Kahler’s disease is the most common malignant plasma cell disorder characterized by clonal proliferation of malignant plasma cells in the bone marrow, monoclonal protein in serum or urine, associated dysfunction of organs (bone and kidney), anaemia and hypercalcemia [1]. The first detailed description of the disease was made in 1884 by the English physician Solly [2], who described the case of a 39-year old patient. Multiple myeloma occurs mostly above 60 years, and its incidence increases with age. The precise cause of myeloma is not known, but many risk factors have been identified notably age, male gender, black skin, exposure to radiations or pesticides, rheumatoid arthritis and obesity [1]. The disease is usually revealed by bone pains, chronic fatigue and fractures of long bones. There is no curative treatment but many drugs can help to prolong survival by 5 to 6 years after the diagnosis [1]. We report a case of multiple myeloma revealed by spinal cord compression and herpes zoster in a 36-year old Cameroonian.

## Patient and observation

Verbal informed consent was obtained from the patient’s next of kin for publication of this case report.

A 36-year-old female farmer with no relevant past medical history was referred to the neurology department for management of a paraparesis of progressive onset since about 6 months, in a context of fever, weight loss and productive cough. Two weeks prior to the consultation, she had herpes zoster skin lesions of right T4 dermatome. Her physical examination on admission revealed altered general state with weight loss, pallor and fever (38.5°C). There was spastic paraparesis with a 2/5 muscle strength in both lower limbs, hypoesthesia with sensory level at T8 dermatome. The middle and inferior abdominal reflexes were absent while the superior abdominal reflexes were present. There were vertebral pains at the level of T8 without deformity or gibbosity. No sign of bowel or bladder dysfunction was observed. Her pulmonary examination revealed diffuse ronchi without crackles and there were no peripheral lymph nodes.

The full blood count showed a normochromic normocytic anaemia with a haemoglobin level of 5.2g/dl. The C - reactive protein (CRP) level was 14mg/l and the Erythrocyte Sedimentation Rate was at 150 mm after the 1st hour. Her HIV serology was negative. The chest X-ray showed bilateral hilar opacities and radiography of the lumbosacral spine revealed sacral demineralization and compression of vertebrae from T12 to L2. On vertebral CT-scan with myelography, there was partial compression of the spinal cord from T6 to T8 with multifocal vertebral osteolysis. CSF analysis showed an increased protein level at 0.75g/l, normal glucose level and no cells.

The initial treatment consisted of a transfusion of 3 pints of packed red cells, an empiric antibiotic therapy with Amoxicillin-Clavulanic acid, Acyclovir, antimalarial treatment and local care of the skin lesions. A week later, there was persistent fever and cough. Three sputum microscopies for Acid Fast Bacilli were negative and blood cultures were sterile. Given the epidemiologic context, weight loss, persistent fever and cough, we started an antituberculous treatment despite the osteolytic lesions which were not quite suggestive of Pott’s disease. The fever disappeared after a few days, but the neurologic deficit did not improve. In order to rule out an osteophylic bone tumour, a thorough examination of the breast was done which was normal and the thyroid was not palpable. The serum level of thyroid hormone (T3 and T4), CA 19-9, CA125 and alpha-fetoprotein were all normal.

The severe bone pains, anaemia, and osteolytic lesions led us to suspect a multiple myeloma despite the patient’s young age. A plain radiography of the skull revealed the typical “punched out” lesions at the vertex and occipital regions. Blood chemistry revealed hypercalcaemia (2.65 µmol/l), hyperproteinemia (101 g/l), hypercreatininaemia (185.8 µmol/l.), hypergammaglobulinaemia and hypoalbuminaemia (23.5g/l) on serum protein electrophoresis. The test for Bence – Jones proteinuria was negative. A bone marrow biopsy revealed a plasmocytosis at 30% thus confirming the diagnosis of multiple myeloma.

The patient was treated with Melphalan 0.2mg/kg/day and prednisone at 40mg daily administered for 7 days every 4 weeks associated with supportive care. There was an initial clinical improvement with normalization of the serum calcium and protein levels within 15 days and healing of the herpes zoster lesions. There was no improvement of the neurological deficit. Better therapeutic regimens including autologous stem cells transplants were considered, but financial and technical constraints did not allow us envisage other protocols apart from the Melphalan/Prednisone association.

Four weeks later, a second dose of Melphalan/Prednisone was administered. However, the patient presented with severe anaemia accompanied by fever and dyspnoea and died a day later despite a blood transfusion, which the patient had initially refused.

## Discussion

Multiple myeloma is a disease of the elderly, the median age at diagnosis being around 70 years [1, 4]. On a total of 14381 patients diagnosed on a30-year period, Kristinsson and collaborators found that 80% of patients were more than 60 years of age and about 1% were aged 40 years or less [3]. Our patient was 36-years old, comparable in age with the first case described in 1888. The cause of the disease is not known, so it is difficult to build up pathophysiological hypothesis for our patient. However, she was found to have several risk factors that could have favoured the development of the disease. She was a black skin farmer who had probably been overexposed to pesticides and other toxic chemical products, which are frequently used for agriculture. Exposure to pesticides is well known as a risk factor for multiple myeloma [1].

The presentation of the disease in this case is atypical. Rather than the bone pains which are the most common presenting features of myeloma in about 58% of cases [1], the patient presented with spinal cord compression due to multifocal collapse of the vertebrae. This type of presentation was found in only 0.5% of 646 cases of myeloma recruited by Cheema and collaborators over a period of 17 years [4]. The patient also presented with herpes zoster lesions on admission. Upon literature review, herpes zoster lesions are described as a complication of antimitotic treatment in patients with refractory multiple myeloma [5], but in our case the patient was not on any specific treatment. Fever is the presenting feature of myeloma in less than 1% of cases [6, 7]. The fever in our patient was probably due to an infection given the context of immune deficiency with herpes zoster lesions on admission, and the improvement of fever on empirical antibiotic treatment. Nevertheless, we can’t exclude myeloma per se as a cause of the fever.

The results of most laboratory tests were similar to those published in the literature for multiple myeloma. The anaemia was normochromic and normocytic and there was no thrombopaenia, which is found in only 10 to 15% of cases [1]. The erythrocyte sedimentation rate was very high in accordance with the abnormal gammaglobulin hypersecretion [8], though the severe anaemia could equally be responsible. The serum calcium was very high and the kidney function test altered. There was a hyperproteinaemia with a gammaglobulin spike on serum protein electrophoresis. The bone marrow biopsy showed a plasmocytosis greater than 10% and a plain radiograph of the skull revealed lytic lesions. The test for Bence-Jones proteins was negative in our patient but this result cannot rule out the diagnosis. The empirical antibiotic treatment was done with a combination of Amoxicillin - clavulanic acid because of its efficiency on the most frequent pathogens (*Streptococcus pneumonia* and *Haemophilus influenza*) seen in lung infections even in patients with myeloma [1].

The European Society of Medical Oncology (ESMO) and the Italian Society of Haematology (ISH) recommend autologous stem cell transplantation, after an initial induction treatment with vincristine, doxorubicin and dexamethasone as the ideal treatment in young patients [9]. However, because it is not accessible in our context, we considered our patient as not a candidate for transplantation and treated her with the association of melphalan and prednisone.

Bisphosphonates reduce osteoclastic bone resorption and thus attenuate bone pains and decrease the risk of pathologic fractures [1, 9]. However this treatment is rare in our setting and was not financially affordable.

Serum beta2-microglobulin (β2M) and albumin levels are the basis for the International Prognostic Index (IPP) which evaluates the prognosis in patients with multiple myeloma [1, 9, 10]. Because of limited finances, the ß2M dosage was not done in our patient. Nevertheless, given the serum albumin level revealed by protein electrophoresis and the rapidly unfavourable outcome of the illness, our patient was probably at stage III of the disease, which corresponds to a life expectancy of about 29 months. Of newly diagnosed patients, about 39% are found in this stage, while 33% are found in stage II and 28% in stage I [10].

We could not identify the exact cause of death in our patient. Taking into consideration the association of fever and severe anaemia, a septic shock is the most probable hypothesis.

## Conclusion

Multiple myeloma also occurs in young individuals. The diagnosis should be considered in all patients with spinal cord compression whatever the age. In resource-limited settings, the association of Melphalan/Prednisone is the treatment of choice.
